# Efficacy of three antimicrobial mouthwashes in reducing SARS-CoV-2 viral load in the saliva of hospitalized patients: a randomized controlled pilot study

**DOI:** 10.1038/s41598-023-39308-x

**Published:** 2023-08-04

**Authors:** Jeniffer Perussolo, Muy-Teck Teh, Nikolaos Gkranias, Simon Tiberi, Aviva Petrie, Maria-Teresa Cutino-Moguel, Nikolaos Donos

**Affiliations:** 1 Centre for Oral Clinical Research, Institute of Dentistry, Barts and The London School of Medicine and Dentistry, Queen Mary University of London (QMUL), London, UK; 2 Centre for Oral Immunobiology and Regenerative Medicine, Institute of Dentistry, Barts and The London School of Medicine and Dentistry, Queen Mary University of London, London, UK; 3 Barts and The London School of Medicine and Dentistry, Blizard Institute, Queen Mary University of London, London, UK; 4 Division of Infection, Newham and The Royal London Hospitals, Barts Health NHS Trust, London, UK; 5 Biostatistics Unit, Eastman Dental Institute, University College London, London, UK; 6 Virology Department, Division of Infection, Barts Health NHS Trust, London, UK

**Keywords:** Viral infection, Oral hygiene, Clinical trials, Population screening

## Abstract

This study aimed to evaluate the efficacy of 3 mouthwashes in reducing severe acute respiratory syndrome coronavirus 2 (SARS-CoV-2) viral load in the saliva of coronavirus disease 2019 (COVID-19) patients at 30 min, 1, 2 and 3 h after rinsing. This pilot study included 40 admitted COVID-19 positive patients (10 in each group). Saliva samples were collected before rinsing and at 30 min, 1, 2 and 3 h after rinsing with: *Group 1*—0.2% Chlorhexidine digluconate (CHX); *Group 2*—1.5% Hydrogen peroxide (H_2_O_2_); *Group 3*—Cetylpyridinium chloride (CPC) or *Group 4* (control group)—No rinsing. Viral load analysis of saliva samples was assessed by Reverse Transcription quantitative PCR. Mean log_10_ viral load at different time points was compared to that at baseline in all groups using a random effects linear regression analysis while for comparison between groups linear regression analysis was used. The results showed that all groups had a significantly reduced mean log_10_ viral load both at 2 (*p* = 0.036) and 3 (*p* = 0.041) hours compared to baseline. However, there was no difference in mean log_10_ viral load between any of the investigated mouthwashes and the control group (non-rinsing) at the evaluated time points. Although a reduction in the SARS-CoV-2 viral load in the saliva of COVID-19 patients was observed after rinsing with mouthwashes containing 0.2% CHX, 1.5% H_2_O_2_, or CPC, the reduction detected was similar to that achieved by the control group at the investigated time points. The findings of this study may suggest that the mechanical action of rinsing/spitting results in reduction of SARS-CoV-2 salivary load.

## Introduction

Coronavirus disease 2019 (COVID-19) outbreak caused by severe acute respiratory syndrome coronavirus 2 (SARS-CoV-2), also commonly known as coronavirus, was declared a pandemic in 2020 by the World Health Organization (WHO) presenting with more than 500 million confirmed cases and 6 million deaths worldwide^1^. COVID-19 is characterized by an unpredictable disease course, ranging from asymptomatic to severe, life-threatening infections^2^. SARS-CoV-2, part of a group of ‘enveloped viruses’ characterized by an outer lipid membrane^3^, has been detected in various clinical specimens such as saliva, throat, nasopharyngeal (NPS), and oropharyngeal (OPS) swabs, and bronchoalveolar-lavage fluid^4^. Angiotensin-converting enzyme II (ACE2), a cell receptor for SARS-CoV which plays an important role in the entry of the virus into the cell, is highly expressed in the oral cavity and oral epithelial cells^5^. A study by To et al.^6^ demonstrated SARS-CoV-2 being detected in 91.7% of the saliva samples obtained from COVID-19 positive patients. In addition, a recent study has further shown that detection rate of SARS-CoV-2 virus in saliva samples can be even higher than that on NPS (93.1% versus 52.5%)^7^. Therefore, saliva from asymptomatic or symptomatic infected subjects could be considered a high-risk route for SARS-CoV-2 infection^8^.

Most measures initially adopted on preventing and limiting transmission of the virus focused on performing good respiratory and hand hygiene, maintaining physical distance, wearing facial masks, and self-isolating. Despite that, different approaches have been proposed as viricidal strategies to target coronaviruses and to interfere with the viral lipid envelope^3,9,10^. Previous studies have suggested that constituents present in oral hygiene products such as toothpastes^11^ and mouthwashes might disrupt the envelope of the virus, an antiviral activity that could inactivate SARS-CoV-2 and potentially dampen transmission of virus^3,10,12^. A recent study investigated the short-term effects of brushing with different toothpastes on the SARS-CoV-2 salivary viral load of patients with COVID-19 showing that immediately after brushing, the use of antimicrobial toothpastes reduced the SARS-CoV-2 salivary viral load^11^.

Mouthwashes have been widely advocated as an adjunctive treatment to mechanical oral hygiene to reduce carious lesions, formation of dental biofilm and gingivitis^13^. Moreover, they have a high level of acceptance among the public due to their ease of use and breath-freshening effect. Different types of mouthwashes exist according to their active ingredients such as cetylpyridinium chloride (CPC), essential oils, chlorhexidine (CHX) or triclosan^13^. Experimental and clinical studies on viral infections have shown that the use of antimicrobial mouthwashes containing povidone-iodine^14^, chlorhexidine gluconate^15,16^, and cetylpyridinium chloride^17^ could reduce viral load. Furthermore, recently it has been demonstrated that the use of CHX, CPC, essential oils or povidone-iodine mouthwashes may reduce SARS-CoV-2 viral load in saliva^16,18,19^. CHX mouthwashes are broadly used as an adjunctive therapy to mechanical plaque removal to reduce oral microorganisms and to prevent oral infections. A report on its in vitro viricidal effectiveness at a concertation of 0.12% showed it may decrease the viral load of enveloped viruses^15^. A recent case study with 2 patients evaluated the viral dynamics in various body fluid specimens including saliva of patients with COVID-19^16^. The findings showed that CHX mouthwash reduced SARS-CoV-2 viral load in the patient’s saliva for 2 h after using the mouthwash, but it increased again at 2–4 h post-mouthwash use^16^. Hydrogen peroxide (H_2_O_2_) formulations have also been suggested to present antiviral activity that could destroy the outer layer of viruses^3,9,10^. Further antimicrobial agents, such as CPC have demonstrated a viricidal activity against susceptible and resistant strains of enveloped viruses by targeting and disrupting the viral envelope. A recent in vitro study assessing the viricidal activity of four mouthwashes demonstrated that the mouthwash formulated with 0.07% CPC showed viricidal effects providing a reduction of Human Coronavirus Strain (HCoV-229E) viral count^18^. In addition, a randomized clinical trial has shown that 0.075% CPC formulated commercial mouth rinse could decrease salivary SARS-CoV-2 levels^20^.

Despite the current findings and promising results of early trials, clinical information regarding efficacy of mouthwashes containing substances such as CHX, H_2_O_2_, and CPC in reducing viral load in the saliva of COVID-19 positive patients and its effects on various SARS-CoV-2 strains is still scarce and contradictory^21–25^. This pilot study tested the hypothesis that mouth rinsing with one of the investigated mouthwashes (0.2% CHX, 1.5% H_2_O_2_ or CPC) would reduce SARS-CoV-2 viral load in the saliva of COVID-19 positive patients at different time points compared to baseline; and that SARS-CoV-2 viral load at each study time point, would differ between rinsing and no rinsing groups. Therefore, the *primary objective* was to evaluate the efficacy of 3 different antimicrobial mouthwashes containing 0.2% Chlorhexidine digluconate, 1.5% Hydroxide peroxide and Cetylpyridinium chloride in reducing SARS-CoV-2 viral load in the saliva of hospitalized COVID-19 positive patients at 30 min, 1, 2 and 3 h after rinsing. The *secondary objective* aimed to compare SARS-CoV-2 viral load in the saliva of COVID-19 positive patients, at different time points, between the 3 mouthwashes (test) and no rinsing (control) groups.

## Material and methods

This was a pilot study conducted in full accordance with the ethical principles of Declaration of Helsinki, revised in 2013 and Good Clinical Practice (GCP) Guidelines. The study protocol was independently reviewed and approved by the National Health Service (NHS) Solihull Research Ethics Committee (Reference number 21/WM/0068; IRAS Number 289334; initial approval 20/04/2021) and registered in a clinical trial registry (ClinicalTrial.gov NCT04723446; 25/01/2021). The study followed the CONSORT checklist for reporting a pilot study (S1 Table).

### Participants and eligibility

Patients were identified in the inpatient wards of Newham University and The Royal London Hospitals (Barts Health NHS Trust, United Kingdom) between April and October 2021, during two peaks of the pandemic and when delta was the prevalent COVID-19 strain^26^. Subjects were eligible for inclusion if they fulfilled the following criteria:

- Males and females, ≥ 18 years old.
- COVID-19 positive confirmed via any diagnostic test and/or presented with COVID-19 clinical symptoms at time of consent.

The exclusion criteria were:

- known pre-existing chronic mucosal lesions e.g., lichen planus or other oropharyngeal lesions, reported by patient or recorded in the existing patient’ medical notes;
- patients intubated or not capable of mouth rinsing or spitting;
- history of head and neck radiotherapy or chemotherapy;
- self-reported xerostomia;
- known allergy or hypersensitivity to chlorhexidine digluconate or any of the mouthwashes constituents;
- other severe acute or chronic medical or psychiatric condition or laboratory abnormality that could increase the risk associated with trial participation or could interfere with the interpretation of trial results and, in the judgement of the investigator, would make the subject inappropriate for entry into the trial.
- inability to comply with study protocol.

The judgment of the investigator was based on the patients’ direct care medical team opinion/recommendation. Before approaching potential participants investigators communicated with patients’ direct care medical team to understand if patient was medically stable and if their participation in the study would not worsen their condition, as well as if they were mentally and physically able to consent and comply with the study protocol.

Potential participants were then approached and provided with the study Patient Information Sheet (PIS) and received explanation about the study. The eligible patients interested in taking part in the study were then invited to sign an informed consent form. Whenever available, COVID-19 test results were confirmed before the patient entered the study. For those patients presenting COVID-19 clinical symptoms at time of consent, positive test status was confirmed within 2 weeks from the date the patient has been consented into the study.

Out of 177 inpatients initially screened, 54 fulfilled the eligibility criteria and were consented for the study. Beyond the initial sample size (n = 40) more participants were recruited into the study, as some of the patients presenting with clinical symptoms at point of consent tested negative for COVID-19 (n = 4) and some of participants initially confirmed as COVID-19 positive by diagnostic test had undetectable SARS-CoV-2 viral RNA in the baseline saliva samples (7; 14.9%). Additionally, after enrolment, 1 participant withdrew consent and 2 were withdrawn from the study by the researcher due to deteriorating medical condition. Characteristics of patients consented but not included (n = 14) in the analysis are presented in the Table S2.

In addition, up to 5 COVID-19 negative participants were recruited and provided signed informed consent as volunteers from, Barts and The London School of Medicine and Dentistry, Institute of Dentistry, Queen Mary University of London to set up the saliva profile of COVID-19 negative patients for analysis.

### Study design and randomization

**Figure 1:**
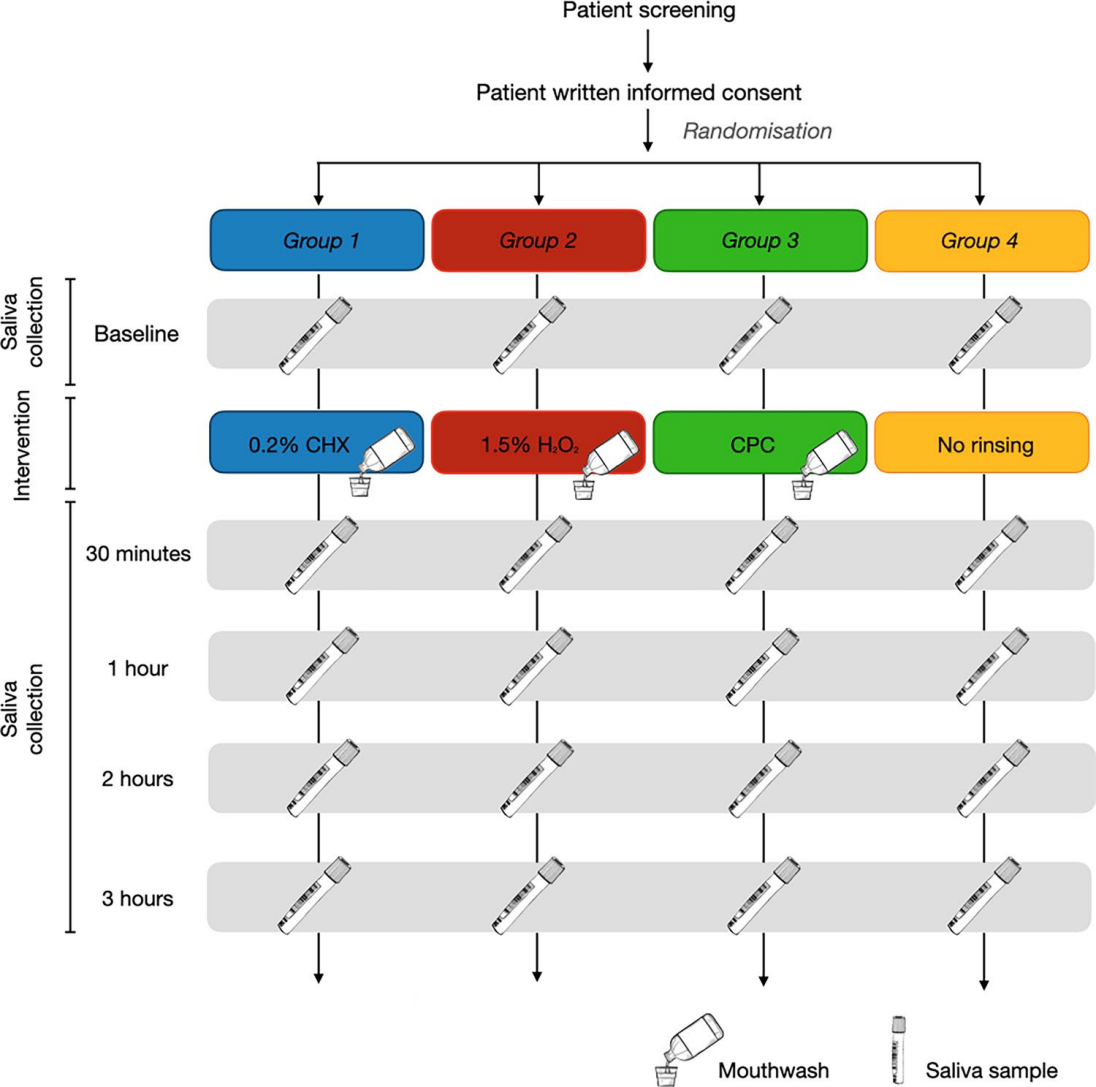
Flowchart with trial profile.

This was a single-blind, parallel-group, randomized controlled pilot study which consisted of a single study visit (Fig. 1). Allocation to intervention group took place via a balanced random permuted block approach. Sets with constant size (4-unit block size) were generated via a computer-based random number generator (http://www.randomizer.org/^27^), ensuring that patients were allocated in balanced blocks to one of the four groups. At the time of enrolment and after informed consent was signed, the study investigator responsible for the intervention allocated each participant as per randomisation to one of the 4 groups:

- *Group 1 (*test group)—0.2% Chlorhexidine digluconate (Corsodyl Alcohol free, GlaxoSmithKline, Brentford, United Kingdom).
- *Group 2* (test group)—1.5% Hydrogen peroxide (Colgate Peroxyl, Colgate-Palmolive, Guildford, United Kingdom).
- *Group 3* (test group)—Cetylpyridinium chloride (Oral-B Gum & Enamel Care, Procter & Gamble, Ohio, United States).
- *Group 4* (control group)—No rinsing (not even water).

### Medical history, oral hygiene habits and demographics

Medical history was obtained as part of the visit, including demographics, oral hygiene habits (e.g., use of mouthwashes and time of last oral hygiene procedure) and concomitant drug use information. Medical history also included relevant results of physical exam, biochemical analysis, diagnostic imaging, COVID-19 test and strain of SARS-CoV-2 variant.

Source documents consisted of patient hospital records (paper or electronic notes) as well as COVID-19 test results/certificates.

### Saliva sample collection

All participants received a sealed self-test kit containing 5 self-collection saliva vials (OMNIgene Oral – OME-505, DNAgenotek, Ottawa, Canada), one for each different time point, and were given instruction to refrain from eating, drinking, chewing gum or performing oral hygiene for at least 30 minutes prior saliva collection (as per manufacturer’s instructions). Participants were then asked to collect a baseline sample of non-stimulated saliva by pooling saliva in the floor of their mouth without swallowing, and then to spit into the sterile vial until the amount of liquid saliva reached the 1 ml indication. At 30 minutes, 1, 2 and 3 hours after rinsing (test groups) or no rinsing (control group) participants were requested to collect saliva following the same recommendations than at baseline. After collecting saliva, patients were instructed to place the vials inside a sealed bag containing absorbent paper in case of opening or breaking of saliva tube. Samples collection was supervised by investigator conducting the study visit.

The self-collection saliva vials contained solution used to inactivate the SARS-CoV-2 virus and stabilise viral nucleic acids at room temperature. After deactivation the saliva samples were stored at room temperature and then transferred to the Blizard Institute, Queen Mary University of London within 3 weeks for viral load analysis. Subsequently, samples were stored in a − 80 °C freezer until destruction in accordance with local guidelines and following the Human Tissue Authority (HTA) Code of Practice.

### Mouthwash use

In the test groups (Group 1–3) patients also received as part of the self-test kit, mouthwash bottles. Immediately after baseline saliva collection, participants were instructed to vigorously rinse their mouth with 10 ml of Corsodyl Alcohol free (Group 1; 0.2% CHX), Colgate Peroxyl (Group 2; 1.5% H_2_O_2_) or Oral-B Gum & Enamel Care (Group 3; CPC) mouthwashes for 1 min, as per randomization. During rinsing, participants were asked to not gargle or swallow the mouthwashes.

Meanwhile, participants in the Group 4 (non-rinsing) were instructed to not rinse their mouth with any solution, not even water. All patients were allowed to drink water as needed during the study period up to 30 min prior each saliva collection.

### Relative quantification of SARS-CoV-2 viral load using RT-qPCR

**Figure 2:**
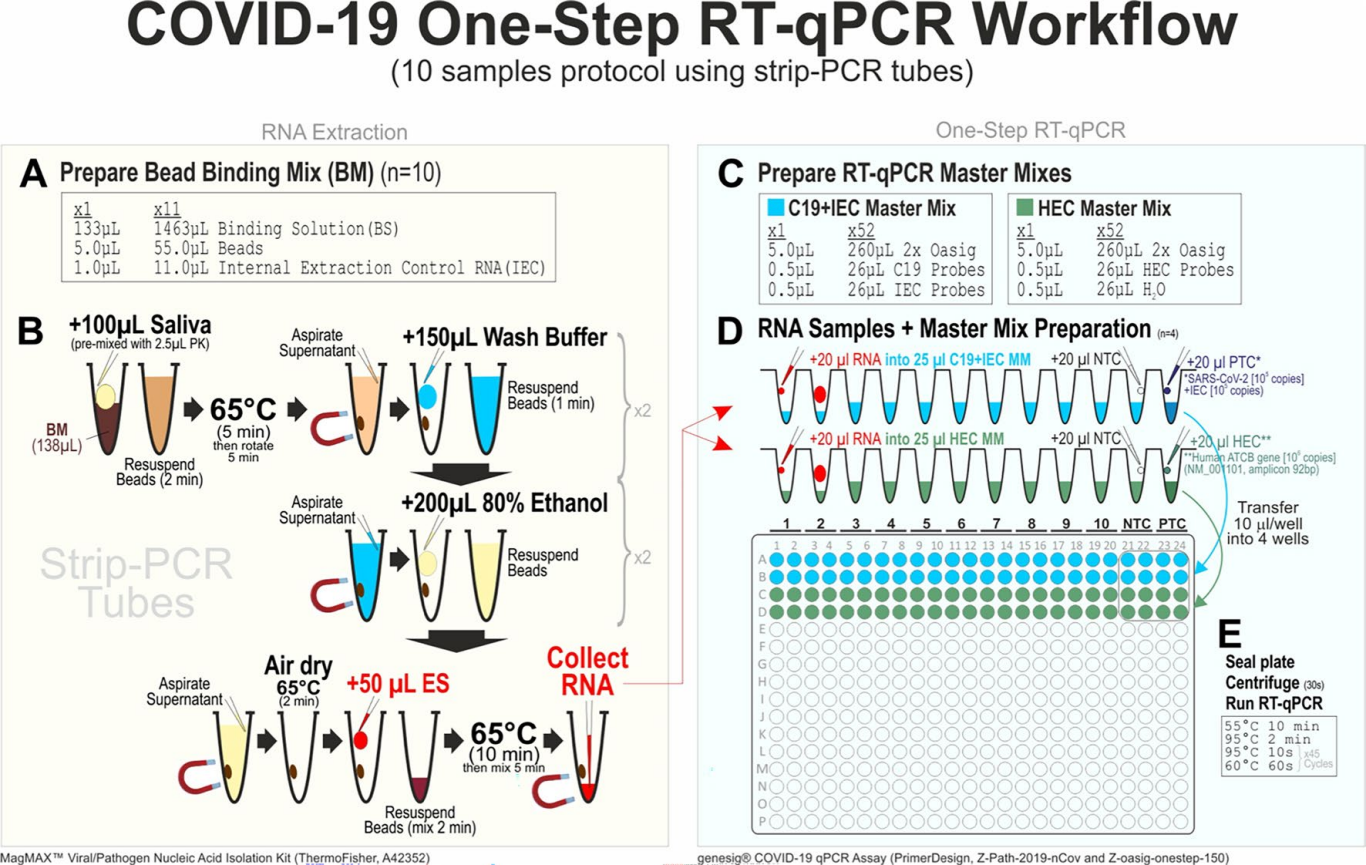
One-step RT-qPCR workflow for quantification of salivary SARS-CoV-2 viral load using MagMAX Viral/Pathogen Nucleic Acid Isolation Kit, genesig COVID-19 qPCR Assay. Figure created using CorelDRAW Graphics Suite 2017 (version 19.1.0.419) https://www.coreldraw.com/en/pages/coreldraw-2017/.

Saliva samples were thawed at room temperature and prior to nucleic acid extraction, saliva samples were spiked with Internal Extraction Control RNA (IEC; an exogenous RNA of rat Phogrin gene, NM_031600, amplicon 98 bp) from the genesig COVID-19 qPCR Assay kit (PrimerDesign Ltd., UK) to verify the successful extraction in case of a negative SARS-CoV-2 result (Fig. 2). Saliva samples provided by healthy volunteers were included as negative controls.

SARS-CoV-2 viral RNA were purified from saliva samples (100–200 µL) using MagMAX Viral/Pathogen Nucleic Acid Isolation Kit (Thermo Fisher Scientific Inc; Cat. A42352) according to manufacturer’s protocol. SARS-CoV-2 viral load were measured using a one-step RT-qPCR kit (genesig COVID-19 qPCR Assay, PrimerDesign, Cat. Z-Path-2019-nCov). As per genesig COVID-19 qPCR assay protocol, SARS-CoV-2 RdRp gene (C19, with FAM 465-510 probe), rat Phogrin gene (IEC, with VIC/HEX 533-580 probe) and a human endogenous control (HEC, with FAM 465-510 probe; ATCB gene, NM_001101, amplicon 92 bp) were measured from each eluted saliva RNA sample in quadruplicate RT-qPCR reactions using a LightCycler LC480 Instrument (Roche Diagnostics, UK). RT-qPCR amplification began with reverse transcription at 55 °C (10 min) followed by Hot-start activation at 95 °C (2 min) prior to 45 cycles of 95 °C (10 s) denaturation, 60 °C (60 s) annealing/extension/acquisition. Every qPCR assay plate included quadruplicate wells of no saliva extraction control, no template control and positive template control for assay quality assurance. The IEC exogenous RNA was used as a positive control for the nucleic acid extraction process. Successful IEC co-detection with SARS-CoV-2 would indicate that PCR inhibitors were not present at a high concentration. Samples with poor IEC amplification (crossing point; Cp > 45) were disqualified as these represented poor RNA extraction (containing inhibitors that interfered with qPCR). HEC gene was measured to confirm successful extraction of a valid biological sample of human origin. Samples with poor HEC amplification (Cp > 45) were disqualified as these indicated samples of insufficient human biological RNA yield. Viral load was calculated from sample Cp/Cq ratio values against a standard curve of Log viral load vs crossing point (Fig. S1). SARS-CoV-2 viral copy number standard curve was determined by performing a tenfold titration series from a stock SARS-CoV-2 template (from genesig COVID-19 qPCR Assay kit) and cross validated using AcroMetrix Coronavirus 2019 RNA positive control kit (Cat. 954,519, Thermo Fisher Scientific Inc., MA, USA) containing two concentrations: a Low Positive (100 copies/µL) and Ultra-Low Positive (500 copies/µL) concentration.

SARS-CoV-2 viral load were normalised against both IEC and HEC to control for RNA quality and human RNA loading, respectively, in each sample. Samples with undetectable viral levels were assigned a Cp value of 45 for calculation.

### Blinding

The investigator responsible for the SARS-CoV-2 viral load analysis was blinded to the study groups/arms.

### Sample size

This pilot study included a convenience sample of up to 40 COVID-19 positive patients, as confirmed by their saliva samples, identified in the wards at the Newham University Hospital and at The Royal London Hospital, both Barts Health NHS Trust sites. The trial was designed considering the number of COVID-19 patients admitted at Barts NHS trust and predictions at time of project development.

### Statistical analysis

Interim analysis of saliva samples collected at baseline, 30 min, 1, 2 and 3 h after mouth rinsing, for the first 2 patients from each study group was performed to identify if a specific mouthwash was not effective in reducing SARS-CoV-2 viral load in the first 1 h and should be excluded from the study. As per interim analysis all mouthwashes were able to reduce viral load, none of them were excluded from the study.

The four measurements for viral load (VL) for each patient at each time point were averaged by taking the mean value which was considered as the unit of measurement. Summary statistics (mean with standard deviation (SD), confidence interval (CI) and median with range) of the viral load at baseline, 30 min, 1, 2 and 3 h after rinsing (test groups) or after no rinsing (control) were determined. Log_10_ viral load data were used for the analysis as the raw data were not normally distributed.

Random effects linear regression analysis, incorporating all time points and an interaction term for times and groups, on the log_10_ viral load data was used to assess the effects of groups (using dummy variables with the control as the reference group) and time points (with the baseline as the reference group).

A linear regression analysis, with the dependent variable being the log_10_ viral load in the saliva at a specific time point and the explanatory variables being the baseline log_10_ viral load and dummy variables for group (0.2% CHX, 1.5% H_2_0_2_, CPC groups and no rinsing [control]), was used to determine if the groups differed in their log_10_ mean viral load after adjusting for the baseline value.

Assumptions of all regression analyses were checked and verified by a study of the residuals. Outliers with a clinical plausibility for exclusion were removed from the analysis (e.g., saliva sample contaminated with food debris, delayed sample collection).

The data was analysed by SPSS (IBM Corp. Released 2020. IBM SPSS Statistics for Windows, Version 27.0. Armonk, NY: IBM Corp) and Stata (StataCorp. 2021. Stata Statistical Software: Release 17. College Station, TX: StataCorp LLC.)*.* Hypothesis tests used a significance level of 0.05.

### Ethical approval

This study was conducted in full accordance with the ethical principles of Declaration of Helsinki, revised in 2013 and Good Clinical Practice (GCP) Guidelines. The study protocol was independently reviewed and approved by the National Health Service (NHS) Solihull Research Ethics Committee (Reference number 21/WM/0068; IRAS Number 289334; initial approval 20/04/2021). Written informed consent was obtained from all individual participants included in the study.

## Results

### Study participants

Forty patients (17 males and 23 females; mean age of 42.6 ± 14.2 years; range 18 – 74 years old), 10 in each group, with detectable SARS-CoV-2 viral RNA in the baseline saliva samples were included in the analysis. Participants characteristics was similar among study groups (Table 1). Out of 200 saliva samples, 6 samples from 3 patients were excluded from the statistical analysis (outliers). Two samples (time points 2 and 3 h; Group 1) were excluded due to presence of food debris, 1 (time point 3 h; Group 2) was collected immediately after patient had food, and 3 samples (time points 1, 2 and 3 h; Group 3) had collection delayed as patient was sleeping.

**Table 1:** Characteristics of study participants (n = 40) according to intervention group.

	Overall	Group 1	Group 2	Group 3	Group 4
		Corsodyl®	Peroxyl®	Oral-B®	Non-rinsing
Gender					
Female (%)	17 (42.5)	3	5	4	5
Male (%)	23 (57.5)	7	5	6	5
Age (years)					
Mean (SD)	42.6 (14.2)	40.5 (15.6)	38.9 (12.4)	43.0 (12.8)	48.0 (16.3)
Median (range)	40 (18–74)	38 (21–73)	39.5 (22–62)	44.0 (18–61)	53.0 (27–74)
Ethnicity					
White (%)	17 (42.5)	3	3	6	5
Asian (%)	8 (20)	3	3	1	1
Black/African/Caribbean (%)	9 (22.5)	4	2	1	2
Mixed or other (%)	4 (10)	0	2	2	0
Not reported (%)	2 (5)	0	0	0	2
Comorbidities					
No (%)	13 (32.5)	3	4	4	2
Yes (%)	27 (67.5)	7	6	6	8
Use of medication during hospitalization					
Antibiotics					
No (%)	22 (55)	5	5	6	6
Yes (%)	18 (45)	5	5	4	4
Tocilizumab					
No (%)	33 (82.5)	9	8	8	8
Yes (%)	7 (17.5)	1	2	2	2
Remdesivir					
No (%)	24 (60)	8	5	5	6
Yes (%)	16 (40)	2	5	5	4
COVID vaccination status					
None (%)	15 (37.5)	4	5	4	2
1 dose (%)	5 (12.5)	2	1	2	0
2 doses (%)	7 (17.5)	2	0	2	3
Not stated (%)	13 (32.5)	2	4	2	5
N. days from positive test					
Mean (SD)	2.20 (2.4)	2.1 (1.9)	2.9 (3.3)	1.7 (2.6)	2.1 (1.7)
Median (range)	1.5 (-5–10)	1.5 (0–5)	1.0 (1–10)	2.5 (-5–4)	1.5 (0–5)
SARS-CoV-2 variant					
Delta (lineage B.1.617.2; %)	28 (70)	8	7	7	6
Delta (sublineage AY.4; %)	2 (5.0)	1	1	0	0
Unable to type or sequencing (%)	8 (20)	1	1	3	3
Not available (%)	2 (5)	0	1	0	1
N. of days from onset of symptoms					
Mean (SD)	8.38 (3.98)	6.5 (2.8)	9.4 (5.7)	8.7 (3.3)	9 (3.4)
Median (range)	8 (3–18)	5.50 (3–12)	9.5 (3–18)	8.5 (4–14)	9 (5–15)

Seventeen (42.5%) participants were White British or any other White background; 9 (22.5%) were Black, Black British, Caribbean, African or any other Black background; 8 (20%) were Asian, Asian British, Indian, or any other Asian background; and 4 were considered mixed or from another (10%) ethnic group (Table 1). Most patients had comorbidities (67.5%) such as diabetes mellitus (25%), overweight or obesity (20%), hypertension (20%) and asthma (17.5%). Details on types of comorbidities among study participants were detailed on Table 2. Regarding use of medication during hospitalization, 45% of patients were taking antibiotics, 40% received an antiviral drug (i.e., Remdesivir), and 17.5% were treated with a medication recently identified as adjuvant in the treatment of COVID-19 pneumonia (i.e., Tocilizumab)^28^, which improves survival and other clinical outcomes.

**Table 2:** Type of comorbidities among the study participants (n = 40), in the study groups. Participants could have more than one comorbidity.

	Overall	Group 1	Group 2	Group 3	Group 4
		*Corsodyl®*	*Peroxyl®*	*Oral-B®*	*Non-rinsing*
	N (%)				
Diabetes	10 (25)	2	3	2	3
Overweight or Obesity	8 (20)	2	1	2	3
COPD or other lung disease	2 (5)	1	0	1	0
Asthma	7 (17.5)	1	2	1	3
Hypertension	8 (20)	3	1	1	3
Cardiovascular disease	4 (10)	2	0	1	1
Renal failure	2 (5)	1	0	1	0
Liver disease	1 (2.5)	0	1	0	0
Other conditions	9 (22.5)	2	2	1	4

Entries are frequency (percentage). COPD—Chronic obstructive pulmonary disease.

All patients had their diagnosis confirmed, as standard of care at the respective hospitals, by reverse transcriptase-polymerase chain reaction (RT- PCR) assays on material collected by combined nasal and throat swab. On average, the time from last COVID-19 positive test result to sample collection was 2 days (ranging from − 5 to 10 days). Meanwhile, the mean time from the onset of symptoms to the study visit and saliva sample collection was around 8 days (ranging from 3–18 days). Most patients included in this study were diagnosed with SARS-CoV-2 variant Delta (lineage B.1.617.2). Among patients included in this study, only 17.5% were fully vaccinated (two doses) while 12.5% had the first dose of COVID-19 vaccine. The remaining patients were unvaccinated (37.5%) or did not have data available on vaccination status (32.5%).

In terms of oral hygiene habits, 57.5% reported using mouthwash as part of their oral hygiene routine at home. The time reported by patient from last oral hygiene (OH) before sample collection ranged from 30 min to 10 days. None of the patients reported any adverse events related to the use of investigated mouthwashes or any of study procedures.

### SARS-CoV-2 viral load

**Figure 3:**
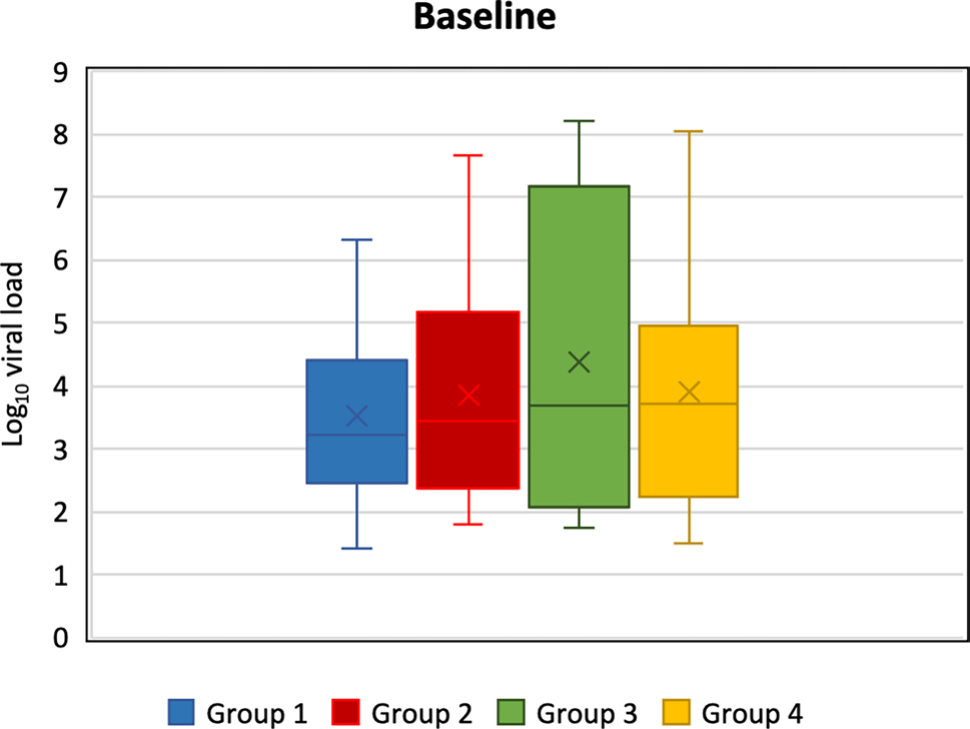
Box plot presenting mean (cross within the box), median (horizontal line within the box), interquartile range, minimum and maximum log_10_ viral load for each group at baseline.

Overall, at baseline mean (SD) and median (min–max) log_10_ viral load were 3.9 (1.9) and 3.4 (1.4–8.2) log_10_, respectively. Boxplots present data on the log_10_ viral load for each Group (1 to 4) at baseline (Fig. 3). Median log_10_ viral load at baseline appeared similar among investigated mouthwashes and non-rinsing group.

**Figure 4:**
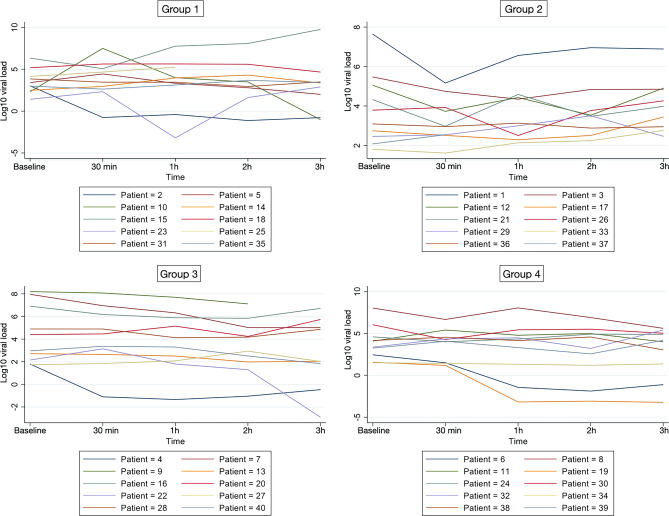
Log_10_ viral load for individual patients in the Groups 1 to 4 at different time points. Graphs created with Stata (StataCorp. 2021. Stata Statistical Software: Release 17. College Station, TX: StataCorp LLC.) Available at https://www.stata.com/products/.

**Figure 5:**
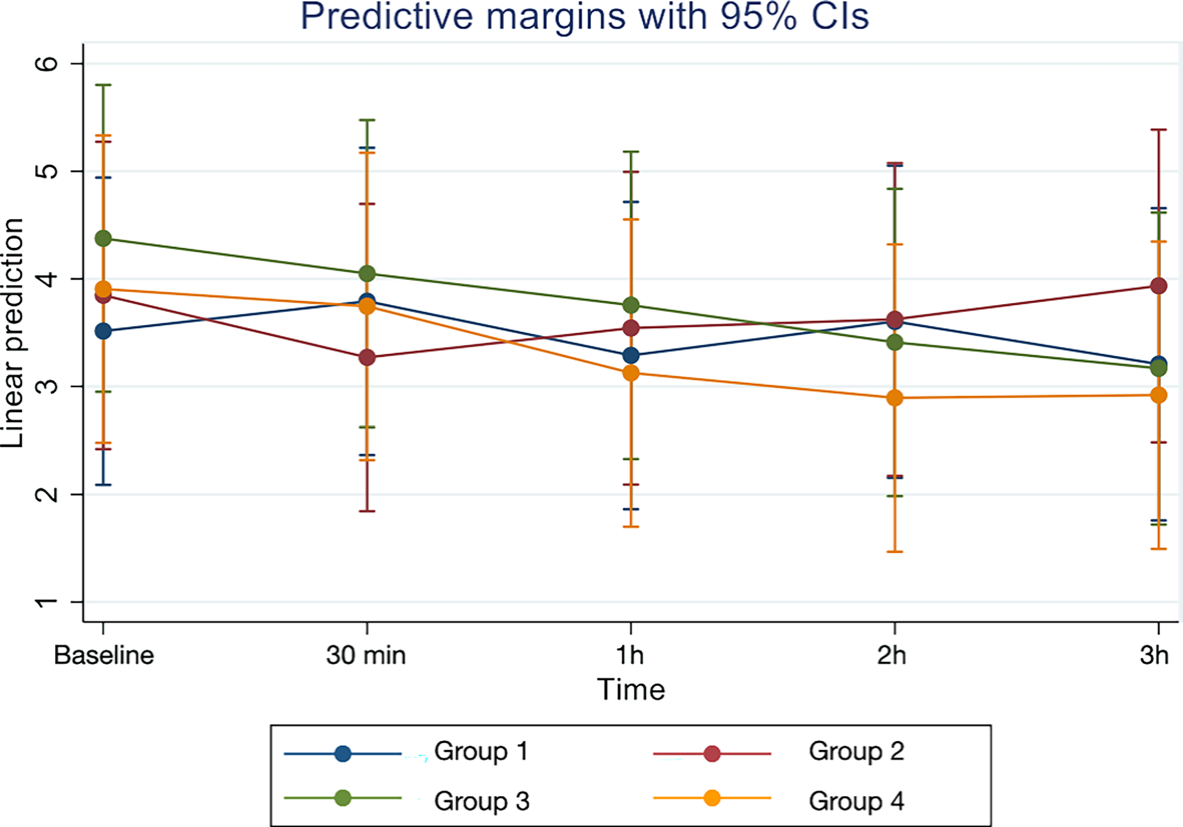
Mean log_10_ viral load for each group with 95% confidence interval (CI) linked at the different time points. Graph created with Stata (StataCorp. 2021. Stata Statistical Software: Release 17. College Station, TX: StataCorp LLC.). Available at https://www.stata.com/products/.

Figure 4 illustrates log_10_ viral load for individual patients in the Groups 1–4 at baseline, 30 min, 1 h, 2 h and 3 h after rinsing or non-rinsing. Participants viral load and responses in the different groups present a high variability. Mean log_10_ viral load for each group with 95% confidence interval (CI) linked at the different time points is presented in Fig. 5.

The results of the random effects analysis showed that on a log_10_ scale, there were no significant interactions between times and groups. This implies that any differences between time points are consistent for each group, and vice-versa. There was no evidence of a difference between the group means but there was a marginally significant difference between the means at 2 h (*p* = 0.036) and at 3 h (*p* = 0.041) when each was compared to the baseline mean. In both cases, the coefficient of the model was negative indicating that the log_10_ mean viral load was reduced at the later time point when compared to that at baseline (Table 3).

**Table 3:** Random effects analysis of mean log_10_ viral load comparing groups (each compared to Group 4, control), time points (each compared to baseline) and interactions.

Log_10_ viral load	Coefficient	*p*-value	95%CI	
			Lower limit	Upper limit
Time point				
30 min	− 0.161	0.738	− 1.107	0.784
1 h	− 0.780	0.106	− 1.726	0.165
2 h	− 1.012	0.036*	− 1.958	− 0.067
3 h	− 0.986	0.041*	− 1.932	− 0.411
Group				
1	− 0.391	0.070	− 2.408	1.627
2	− 0.058	0.954	− 2.076	1.958
3	− 0.470	0.648	− 1.548	2.487
Time point: Group				
30 min: Group 1	0.438	0.520	− 0.898	1.775
30 min: Group 2	− 0.416	0.542	− 1.753	0.921
30 min: Group 3	− 0.166	0.808	− 1.503	1.170
1 h: Group 1	0.554	0.416	− 0.783	1.890
1 h: Group 2	0.476	0.494	− 0.887	1.839
1 h: Group 3	0.159	0.815	− 1.177	1.496
2 h: Group 1	1.101	0.113	− 0.259	2.462
2 h: Group 2	0.789	0.257	− 0.574	2.152
2 h: Group 3	0.465	0.946	− 1.290	1.383
3 h: Group 1	0.679	0.328	− 0.682	2.039
3 h: Group 2	1.074	0.122	− 0.289	2.437
3 h: Group 3	− 0.222	0.748	− 1.582	1.137

* Significant at 5% (*p* < 0.05).

Linear regression analysis at each time point was used to determine if the mean log_10_ viral load of each of the Groups 1, 2 and 3 differed significantly from that of the control group (reference category) at 30 min, 1, 2 or 3 h after rinsing, after adjusting for the baseline log_10_ viral load (Table 4). However, there were no significant differences between the means of any of the rinsing (test) groups compared to the control group (non-rinsing) at any time point (30 min, 1, 2 and 3 h). The same was observed when comparing mean log_10_ viral load of each of the Groups 1, 2 and 3 (test groups) at the different time points after adjusting for the baseline log_10_ viral load.

**Table 4:** Regression analysis used to determine if Groups (1–3) differ in their mean log_10_ viral load from that of the control group (4) at the different time points, after adjusting for the log_10_ viral load at baseline.

Model	Coefficient	*p*-value	95% CI for β	
	b		Lower bound	Upper bound
30 min				
Constant	0.711	0.259	− 0.546	1.967
Group 1	0.351	0.568	− 0.886	1.588
Group 2	− 0.429	0.485	− 1.663	0.805
Group 3	− 0.061	0.921	− 1.300	1.178
1 h				
Constant	− 1.305	0.085	− 2.801	0.190
Group 1	0.607	0.405	− 0.857	2.070
Group 2	0.382	0.608	− 1.118	1.882
Group 3	0.096	0.895	− 1.369	1.561
2 h				
Constant	− 0.992	0.168	− 2.424	0.440
Group 1	1.052	0.147	− 0.388	2.492
Group 2	0.714	0.319	− 0.721	2.150
Group 3	0.049	0.944	− 1.353	1.451
3 h				
Constant	− 1.290	0.158	− 3.108	0.529
Group 1	0.668	0.448	− 1.101	2.436
Group 2	0.989	0.262	− 0.773	2.750
Group 3	0.203	0.816	− 1.964	1.558

*Significant at 5% (*p* < 0.05).

The reference category is Group 4 (control) for each group comparison.

## Discussion

This pilot study provided important information on the impact of three different mouthwashes containing 0.2% Chlorhexidine digluconate (CHX), 1.5% Hydroxide peroxide (H_2_O_2_), and Cetylpyridinium chloride (CPC) on SARS-CoV-2 viral load, mainly variant Delta, in the saliva of hospitalized COVID-19 patients. The present study suggested a marginally significant reduction in the mean SARS-CoV-2 log_10_ viral load in the saliva, both at 2 and 3 h after rinsing (test) and non-rinsing (control) compared to baseline. However, there was no evidence of a difference in the mean log_10_ viral load changes between any of the investigated mouthwashes and the control group. This agrees with other studies by Chaudhary et al.^29^ and Ferrer et al.^30^ in which the use of 1% H_2_O_2_, 0.12% CHX, or 0.07% CPC mouthwashes showed a reduction in viral load up to 2 h after mouth rinsing but did not present an advantage to distilled water or saline group (control group). These findings may suggest that mouth rinsing or just spitting alone (as in the present study) may have facilitated “clearance” and contributed to lower viral load in the saliva even in the control groups. At the same time and in contrast to our findings and the previous studies mentioned, a recent pilot trial has shown that mouthwashes containing 0.075% CPC plus 0.28% zinc lactate (CPC + Zn) or 1.5% hydrogen peroxide (H_2_O_2_) were able to reduce viral load by 20.4 ± 3.7 and 15.8 ± 0.08-fold, respectively, immediately after rinsing^12^. While the CPC + Zn group maintained a > twofold reduction after 1 h, H_2_O_2_ mouthwash was capable to decrease SARS-CoV-2 viral load up to 30 min after rinsing. Moreover, 0.12% CHX mouthwash presented a > 2-fold reduction at immediately after, 30 and 60 min after OH (2.1 ± 1.5-, 6.2 ± 3.8-, and 4.2 ± 2.4-fold reductions, respectively)^12^. However, comparisons between control and test groups were not performed. Thus, it is not possible to confirm if the changes identified were related to the antimicrobial activity of the mouthwashes used or its “clearance” effect through the rinsing mechanical action. The heterogeneity of results encountered by different studies in the literature could be explained by many factors such as sample size, absence of a control group for comparison, lack of a longer experimental period, frequency of mouthwash use and type of SARS-CoV-2 variant.

To the best of authors’ knowledge this is the first clinical study to investigate the efficacy of different mouthwashes in reducing viral load in the saliva of COVID-19 patients with delta variant. Among the participants included in the present investigation, delta SARS-CoV-2 strain was the most prevalent whilst in other clinical studies the type of SARS-CoV-2 strain investigated was not disclosed. Previous studies have found that lower concentrations of CPC (10–40 μg/mL) presented anti-SARS-CoV-2 effects in many strains (Wuhan, Alpha, Beta, and Gamma). Meister et al.^31^ also found that in vitro different SARS-CoV-2 strains could be efficiently inactivated with CHX and other commercially available mouthwashes. However, it is still unclear how different SARS-CoV-2 strains can be affected by mouthwash use and if results can be extrapolated to distinct variants.

In the present study, SARS-CoV-2 viral load was assessed by RT-qPCR and was normalized to two reference genes; one external rat gene and another a human actin gene to consider extraction variation in human material. In previous studies^20,32^, raw cycle threshold (Ct) data generated by RT-qPCR assays was used as viral load indicators. The use of Ct values as an indirect method of arbitrarily quantifying the viral load may lead to misinterpretation of results^33^. To accurately measure the number of viral copies in the original sample, the amount of biological material retrieved should also be considered. Thus, the application of a normalization method for the Ct values, by using a reference gene as in our study, is critical for the interpretation of RT-qPCR results^33,34^. A recent study comparing raw Ct values and ΔCt normalized to a reference gene found that ΔCt values provide better accuracy and improve the interpretation of RT-qPCR studies that use SARS-CoV-2 viral load^33^. In the same study it was also shown that nasopharyngeal swab (NPS) samples initially considered to have different viral loads by raw Ct comparison, had the same viral load when a reference gene was taken into consideration for the analysis. It is also important to highlight that although RT-qPCR protocol can detect presence of viral genetic material in a sample, is not able to distinguish between infective and non-infective dead/ non-viable viral particles^35^. The statistical selection criteria by different studies when analysing the outcome of RT-qPCR data may also have important implications in the interpretation of results. A study by Dalman et al. (2012) has suggested that different significance level along with the different fold changes cut-offs can give very distinct results, that may interfere with data interpretation. Although some studies have demonstrated that a specific fold change would be suggested to be clinically relevant, there is no evidence demonstrating that this reduction in the viral load could also decrease the risk of infection and consequently contribute to preventing transmission^11^.

The variability in the viral load, also observed in the control group of the present investigation, may be explained by naturally occurring changes in the shedding of viruses from other body niches like the nasopharynx. This information can be supported by the fact that persistent viral genetic material has been identified in upper respiratory samples weeks after the COVID-19 symptoms have disappeared. SARS-CoV-2 replicates abundantly in upper respiratory epithelia, where ACE2 is expressed^36–38^. Although a mouthwash use may reduce viral load in the saliva of COVID-19 patients, virus will still replicate in the upper respiratory epithelia which may consequently restore the amount of virus in the saliva. In addition, a study presenting a within-host modelling of viral load dynamics in the upper respiratory tract (URT), has shown a wide variation in the viral load between individuals, at different time points from symptoms onset^39^. Furthermore, episodes of coughing in the period of sample collection may influence the saliva viral load^29^.

Despite the mouthwashes used in the current study did not contain ethanol on their formulation, other commercially available mouthwashes can also be formulated with ethanol which could have contributed for reducing viral load. However, the majority of evidence available on the efficacy of mouthwashes in reducing viral load did not provide detailed information on the formulation of the mouth rinses investigated. In a study by Biber et al.^40^ where patients rinsed and gargled using a mouthwash (Listerine) with or without ethanol (Orbitol), nucleocapsid viral gene was detected in 42% and 50% of samples, respectively, suggesting a similar effect of both mouthwashes. Thus, it is still unclear how the different formulations and components present in the mouthwashes could have influenced the results.

SARS-CoV-2 viral RNA was detected in the saliva samples from 85% (45/53) of our patients diagnosed with COVID-19. On average, the mean time from the onset of symptoms to the study visit and saliva sample collection was around 8 days (ranging from 3 -18 days). The median incubation period from infection with SARS-CoV-2 to onset of symptoms is approximately 5 days with viral load reduction with recovery. Despite that, hospitalised individuals presenting with more severe illness are more likely to exhibit longer shedding of potentially infectious virus. Although the number of days from onset of symptom could have influenced the salivary viral load of the patients initially consented in this study, viral RNA was still detected in most of the saliva samples. Similarly, to the present investigation, previous studies were able to identify SARS-CoV-2 in 83.6–91.7% of the saliva samples obtained from COVID-19 positive patients^40^. A recent study has also shown that detection rate of SARS-CoV-2 virus in saliva was even higher than that on nasopharyngeal swabs (NPS) (93.1%; 149/160 versus 52.5%; 84/160; *p* < 0.001)^7^. This demonstrates that saliva is a reliable tool for detection of viral gene and may be considered a diagnostic option for SARS-CoV-2 detection and an alternative to nasopharyngeal swab^7^.

The results from the present study should be considered in the context of some limitations. This study included critically ill hospitalized COVID-19 patients. Although participants were instructed and refrained from eating, drinking, or performing oral hygiene for at least 30 min prior each saliva sample collection, it was challenging to control and restrict the intake of drinks and food during the whole period of the study visit. In addition, most patients were on oxygen via nasal cannula which may have contributed to the reduction of the salivary flow. The authors also acknowledge that the inclusion of an additional group, who would rinse their mouth with saline or distilled water, would have allowed for a better understanding of the mechanical effect of rinsing on the outcomes. However, including the comparison of mouthwash use to not rinsing the mouth at all was deemed important to gather information on the saliva viral load specifically for those patients who did not perform any oral hygiene procedures. Furthermore, although in the present study patients taking antivirals or drugs that could have influenced salivary viral load were not excluded, the number of subjects taking medications were evenly distributed among groups and were unlikely to influence main results.

Despite the current findings, the direct potential benefit of reducing SARS-CoV-2 load in the saliva of COVID-19 positive patient, in terms of disease transmission and patient wellbeing, is still unknown and needs to be further investigated. In future studies, the use of different or novel antimicrobial oral solutions could be established as a possible approach to either reduce transmission of coronavirus in the early stages of infection, especially among health professional (i.e., dentists, surgeons, and anaesthetists) or reduce viral load to potentially restrict virus transmission preventing future disease outbreaks. Additionally, it is not known if the reduction of salivary viral load could contribute to alleviate patients’ symptoms. Irrespective of its effect on SARS-CoV-2 salivary load, it is important to note the significance of maintaining good oral hygiene and care for patients diagnosed or suspected to have COVID-19 during and after infection resolves. Previous studies have demonstrated the importance of oral hygiene in overall health, particularly in hospitalized patients, and its association with various systemic conditions including respiratory and cardiovascular disease. Good oral hygiene practices, such as regular brushing, interdental cleaning, and mouthwash use, can contribute to maintain oral health and prevent oral infections, which can impact individuals overall well-being.

## Conclusion

Taking into consideration the findings and limitations of the present pilot study, it may be concluded that:

(i) The mouthwashes containing 0.2% CHX, 1.5% H_2_O_2_, or CPC did not show difference over the control group in reducing SARS-CoV-2 viral load in the saliva of hospitalized COVID-19 patients at the investigated time points.
(ii) The marginally significant reduction in the average viral load observed in all study groups both at 2 and 3 h compared to baseline, may suggest a mechanical effect/action attributed to rinsing or spitting.
(iii) Further, evidence from well-designed randomised clinical trials is required to fully understand the impact of oral hygiene and oral care products on viral load, disease transmission and patient wellbeing.

### Supplementary Information

Supplementary Information.

## Data Availability

The datasets used and/or analysed during the current study are available from the corresponding author on reasonable request.
